# Host Cellular Receptors for the Peste des Petits Ruminant Virus

**DOI:** 10.3390/v11080729

**Published:** 2019-08-08

**Authors:** Meera Prajapati, Niyokwishimira Alfred, Yongxi Dou, Xiangping Yin, Raju Prajapati, Yanmin Li, Zhidong Zhang

**Affiliations:** 1 State Key Laboratory of Veterinary Etiological Biology, Lanzhou Veterinary Research Institute, Chinese Academy of Agricultural Sciences, Xujiaping 1, Lanzhou 730046, China; 2 Deutsche Gesellschaft für Internationale Zusammenarbeit (GIZ) GmbH, P.O. Box 1457, Kathmandu, Nepal; 3 CAAS-ILRI Joint Laboratory for Ruminant Disease Control, Lanzhou Veterinary Research Institute, Chinese Academy of Agricultural Sciences, Xujiaping 1, Lanzhou 730046, China

**Keywords:** *Morbillivirus*, Nectin-4, PPRV, SLAM

## Abstract

Peste des Petits Ruminant (PPR) is an important transboundary, OIE-listed contagious viral disease of primarily sheep and goats caused by the PPR virus (PPRV), which belongs to the genus *Morbillivirus* of the family Paramyxoviridae. The mortality rate is 90–100%, and the morbidity rate may reach up to 100%. PPR is considered economically important as it decreases the production and productivity of livestock. In many endemic poor countries, it has remained an obstacle to the development of sustainable agriculture. Hence, proper control measures have become a necessity to prevent its rapid spread across the world. For this, detailed information on the pathogenesis of the virus and the virus host interaction through cellular receptors needs to be understood clearly. Presently, two cellular receptors; signaling lymphocyte activation molecule (SLAM) and Nectin-4 are known for PPRV. However, extensive information on virus interactions with these receptors and their impact on host immune response is still required. Hence, a thorough understanding of PPRV receptors and the mechanism involved in the induction of immunosuppression is crucial for controlling PPR. In this review, we discuss PPRV cellular receptors, viral host interaction with cellular receptors, and immunosuppression induced by the virus with reference to other Morbilliviruses.

## 1. Introduction

Peste des Petits Ruminant (PPR), also known as sheep and goat plague, is a highly contagious fatal viral disease of sheep and goats caused by the PPR virus (PPRV), which represents one of the most economically important animal diseases in areas whose economy relies on small ruminants. Currently, it is endemic in Asia and Africa; however, it has crossed into the territories of 70 countries in the last two decades, affecting 30 million animals every year globally [1]. The primary hosts of PPRV are goats and sheep, but the degree to which different species of animals develop clinical disease varies. Goats seem to be more susceptible than sheep [2]. Additionally, an experimental study where both sheep and goats were administered a virulent strain of PPRV showed that sheep displayed milder clinical signs compared to goats, thereby showing the evidence of the greater susceptibility of goats compared to sheep [3]. Interestingly, cattle, buffalo, camel and pigs can develop subclinical infections, but do not excrete the virus [4,5]. Overall, the morbidity and mortality rate may reach 100% and 90%, respectively [6]. Although transmission occurs via aerosol droplets to the respiratory system, the virus mainly localizes in the regional lymph nodes (pharyngeal and mandibular) and tonsils, resulting in lymphopenia [7]. Similarly, it has been demonstrated that the prime replication site of PPRV is the tonsillar tissue and lymph nodes, draining to the site of inoculation but not within the epithelial cells of the respiratory mucosa, suggesting that the virus attaches to the immune cells of the respiratory mucosa, which is transported to the lymphoid tissues where primary replication occurs [6]. The disease then progresses to viraemia, disseminating the virus to all visceral lymph nodes, bone marrow, the spleen, and the mucous membranes of both the respiratory and digestive tract [7]. There is no carrier status in PPR, like in Rinderpest, but infected animals may transmit the disease during the incubation period [8].

For the entry of the virus into target host body, receptor-mediated endocytosis is required, which begins with the initial attachment of the virus to cell-surface receptors. Two cellular receptors have been implicated as putative receptors for PPRV to enter the target cells in vivo. However, detailed information on virus interactions with these receptors and its impact on the host immune response is still required. A thorough understanding of the role of the receptors in PPRV infection and the mechanism involved is crucial for controlling PPR. In this review, we focused on the molecules which have been cited as possible receptors for PPRV and discuss the impact of the interaction of cellular receptors with the virus on the disease pathogenesis and host immune response to the virus, with reference to other Morbilliviruses.

## 2. PPRV Genome Organization

The PPR virus is classified under the genus *Morbillivirus* of the family Paramyxoviridae, and is an enveloped, linear single stranded, negative sense RNA virus that is structurally and genetically related to other members of this genus, such as the Rinderpest virus (RPV) of cattle and buffaloes, Measles virus (MeV) of humans, Canine Distemper virus (CDV) of dogs and some wild carnivores, and the Morbilliviruses of aquatic mammals [9]. Previously, PPRV was regarded as a variant of RPV adapted specifically for goats and sheep; however, now, the two viruses are known to be completely different, though closely related antigenically.

The PPRV genome is 15,984 nucleotides long [10], and the size of the virion ranges between 400 and 500 nm, which is larger than RPV (300 nm) [10,11,12]. All the genes follow the order of 3’ N–P/C/V–M–F–H–L 5’ [10], where each gene is separated by an intergenic region of variable length [13]. It encodes two non-structural proteins (V, C) and six structural proteins Fusion (F) protein, Haemaglutinin (H) protein, the matrix protein (M), the Nucleoprotein (N), and the Phosphoprotein (P), which form the polymerase complex in association with large proteins (L) [14,15,16,17]. The M protein, located inside the envelope, stabilizes the virus structure and surrounds the viral RNA, which is associated with the N protein. Untranslated regions at the 3’ and 5’ ends of both genomic and antigenomic RNA serve as promoters, which play a crucial role during transcription and replication of Paramyxoviruses [18]. During virus budding, the viral envelope is derived from an infected cell membrane and studded with glycoprotein peplomers consisting of F and H proteins. The H and F proteins are the spike glycoproteins, which are inserted into the viral envelope. The matrix protein M serves as a link between the glycoproteins and ribonucleo-protein complex (RNP), mainly containing the viral RNA and the N protein surrounding it. In the virions, P, N, and L proteins constitute the nucleocapsid, which encloses the viral RNA. There is only one single serotype of PPRV. However, phylogenetic analysis based on the small region of the N/F gene showed that the virus can be classified into four distinct lineages (I–IV). In general, Lineages I and II are present in West-Africa, Lineage III in Arabia and East Africa, and Lineage IV in Asia and the Middle East [19].

## 3. PPRV Host Cellular Receptors

The signaling lymphocyte activation molecule (SLAM) (also known as CD150) and the cell adhesion molecule Nectin-4 (also known as poliovirus receptor-like protein 4, PVRL-4) are major cellular receptors required for PPRV to attach to cells [20,21]. In addition to SLAM and Nectin-4, the membrane cofactor protein (CD46/MCP) is also known to be the cellular receptor for the vaccine and laboratory passaged strains of MeV only [22]. SLAM/CD150 mediates infection of immune cells and dissemination of the virus, whereas Nectin-4 mediates infection of epithelial cells and is considered to play major role in virus transmission. Recently, Yang et al. demonstrated PPRV entry into caprine endometrial epithelial cells (EECs) via the caveolae-mediated endocytosis pathway, which is different from the entry mechanism into caprine foetal fibroblast cells [23]. This finding implies that, in addition to direct fusion at the cell surface, PPRV might penetrate goat epithelial cells through endocytosis.

### 3.1. Signaling Lymphocyte Activation Molecule (SLAM/CD150)

SLAM/CD150 is a major cellular receptor of Morbilliviruses [24]. Human SLAM was first identified as a lymphoid cellular receptor for wild type MeV by Tatsuo and colleagues [25], through screening of a cDNA library of B95a cells. Subsequently, it was also demonstrated that CDV and RPV use dog and cow SLAM as a receptor, respectively [24]. Likewise, PPRV has the propensity to use SLAM as a receptor. Pawar et al. reported that SLAM is one of the (co)receptors for PPRV [26]. On the other hand, the SLAM genes of goat, sheep, cattle, and buffalo have presented with 96.3–98.5% and 92.9–96.8% identities at the nucleotide (nt) and amino acid (aa) level by sequence analysis, respectively [27]. Considering the homology of the sequences through comparative analysis of the amino acids, caprine SLAM was found to share a high degree of homology with sheep SLAM (96.8%), followed by cattle (93.5%), buffalo (92.9%), dog (70.2%), and human (62.8%) SLAM [27]. These studies suggest that the SLAM gene has at least a few significant amino acid differences, which might be the reason for the species specificity of Morbilliviruses. Moreover, this receptor specificity leads to a species barrier for Morbilliviruses. Above all, the adaptation of Morbilliviruses to new species might be due to the presence of conserved amino acids in different animal species, which needs to be explored further. Ohishi et al. discovered the eight key amino acid residues in the goat SLAM V domain which affect host-virus specific binding, and are also completely conserved in sheep, cattle, and buffalo [28].

SLAM/CD150 is a glycoprotein grouped under the CD2 subset of the immunoglobulin super family of surface receptors. This CD2 family of proteins acts as both adhesion molecules and modulators of the immune response [29,30,31]. Considering the structure of mature human SLAM, it has 217 amino acids (aa), an extracellular domain (ECD) with two Ig-like domains, a 21 aa transmembrane segment, and a 77 aa cytoplasmic domain with three immunoreceptor tyrosine switch motifs [32]. CD150/SLAM is expressed on activated T cells, memory T cells, T cell clones, immature thymocytes, mature dendritic cells, activated monocytes, primary B cells, and also on Epstein-Barr virus (EBV) transformed B cells (B95a cells) [33]. The ectodomains of the SLAMF (SLAM family) receptors are homophilic or self-ligand receptors, except SLAMF2 (CD48), which uses both SLAMF4 (CD244) and CD2 as its counter ligands. CD150 has a low affinity for binding to itself (Kd ≥ 200 µM), however, the binding avidity increases due to the redistribution or clustering of molecules after cell activation.

In the case of MeV, the human CD150 V domain is sufficient to interact with the MeV-H protein and allow MeV entry [34]. Other regions of human CD150, including the C2 domain, TM and CY, do not play any role in binding of the virus with the host cell [35]. Substantial evidence has shown that MeV binds with the receptor SLAM at histidine 61 and its adjacent amino acid residues at positions 60 (Isoleucine) and 63 (Valine) [35]. In addition, amino acid residues at positions (Lysine K) 58, 59, and 61 also appear to contribute to the receptor function of human Cd150. Residues at position m60, 61, and 63 were later also found to be important for the function of SLAM as a receptor for CDV. Even though mouse CD150 shares a 60% identity at the amino acid (aa) level with human CD150, it cannot act as a receptor for MeV [36,37]. However, the amino acid substitutions at the positions 60, 61, and 63 in human type residues may make mouse CD150 act as an MeV receptor [38]. Furthermore, it has been reported that MeV, CDV, and RPV can use the CD150 of non-host species with lower affinity [38]. The interaction of SLAM and Morbillivirus is not only the first contact point between the virus and host, but is also the important cause of the pathological changes in the host organism and the resulting clinical signs [39]. SLAM has a high affinity for H protein, which can induce downregulation of SLAM in cells either by expressing or by coming in contact with it [32]. Interestingly, H-protein interacts with CD150 through sequential H-protein conformational changes [40].

SLAM/CD150 are expressed on primary peripheral blood lymphocytes. The CD4+ and CD8+ memory T cells express higher levels of CD150 than naïve cells [41,42,43]. B cells have been shown to contain fewer cells that express CD150, but the frequencies of CD150+ cells were higher in naïve B cells than in the memory subsets, in contrast to T cells [42]. The study by Laksono et al. [42] indicated that both naïve and memory B cells, along with several other antigen-experienced lymphocytes, are target cells of MeV, and its depletion leads to immunosuppression. However, in PPRV, the infection results in T cell depletion and impairment of T cell activation but a significant increase in B cells, suggesting a T cell independent B-cell activation response [44,45]. A previous study revealed that IFIT3 and TRIM56 are early innate immune molecules following PPRV infection [46]. The immunosuppression caused by PPRV can be characterized by damage in the lymphoid organs, apoptosis in peripheral blood mononuclear cells in vitro [47], and extensive necrotic lesions in the spleen, lymph nodes, thymus, and Peyer’s patches [6,48]. On the other hand, CD8+ T cells have been found to increase after seven days post challenge in both vaccinated and unvaccinated goats [45], while CD4+ cells increased in unvaccinated sheep [44]. These results imply that there is T cell deactivation during the early phase of infection, providing the opportunity for the virus to multiply and spread.

### 3.2. Nectin-4/PVRL4

In the beginning, it was thought that even though SLAM was identified as a cellular receptor for Morbilliviruses, the infection of MeV in SLAM negative epithelial cells of the trachea, bronchial tubes, lungs, oral cavity, pharynx, esophagus, intestines, liver, and bladder indicated that entry of MeV is mediated by other cellular receptors [49]. Until 2001, the putative receptor in epithelial cells was termed EpR, and later on it was discovered to be Nectin-4/PVRL-4 [50,51]. The V domain of Nectin-4 binds strongly to MeV H protein, similar to the SLAM molecule, where its V domain interacts with the MeV H protein [50,52]. Subsequently, Nectin 4 was also shown to be an epithelial receptor for the PPR virus and Canine Distemper virus [20,53].

Nectin-4, or PVRL4, is a member of the poliovirus receptor-like proteins (PVRLs), consisting of 510 amino acid transmembrane proteins and with a molecular weight of 66 kDa. The term “necto” in latin means “to connect”, which suggest the role of nectins in Ca^2+^ independent cell-cell adhesion [54]. Similar to SLAM, nectin molecules have immunoglobulin-like domains. In fact, they consist of three Ig-like ectodomains (an N terminal V-type which mediates ligand binding and two C2 domains), a transmembrane region, and a cytoplasmic tail [54,55]. Their V domain plays a key role in homotypic or heterotypic interactions with Nectin-1, and the C2 domains enhance the affinity of these interactions [55,56,57]. Nectin molecules are expressed broadly in tissues, including haematopoetic, neuronal, endothelial, and epithelial cells, except for Nectin-3 which displays a more restricted expression [58,59]. Nectin-4 in humans is mainly expressed in the placenta, whereas in mice it is expressed in the brain, lung, testes, and embryos [60]. Nectin-4 has also been detected in the dog brain, suggesting the infection of neurons by CDV and its neuropathogenicity via Nectin-4 [61]. Additionally, this study discovered the variation in the distribution pattern of Nectin-4 in the central nervous system (CNS) of dogs and humans. Nectin-4 has been reported in the ependymal cells, epithelia of the choroid plexus, meningeal cells, neurons, granular cells, and purkinje’s cells of dogs, but has not been detected in the human brain [62]. On the other hand, the distribution pattern of Nectin-4 expression in the epithelial tissues of dogs was found to be similar to that of humans [51,63]. Nectin-4 is highly conserved between various species, which removes the need for receptor adaptation by the virus, unlike in SLAM [20,64]. Subsequently, the VeroNectin-4 cell line was developed for isolation and titration of PPRV, which was found to be more sensitive than VeroDogSLAM cells [64]. The extracellular domain of mouse Nectin-4 shares 92% of its identity with its human counterpart [60]. The nectin family proteins are novel regulators of cellular activities, including cell polarization, differentiation movement, proliferation, and survival [54,65]. Nectin-4 has been shown to be upregulated in human brain endothelial cells by MeV infection [66]. In humans, MeV initially multiplies in T cells, B cells and dendritic cells expressing SLAM/CD150, and then disseminates to distant sites of the host including the kidneys, gastrointestinal tract, liver, and airway epithelial cells of the respiratory tract. Airway epithelial cells seem to be infected through the basolateral surface (adherens junction), expressing Nectin-4 presumably through contact with infected immune cells [50,67,68] and subsequently release of the virus into the environment. Hence, Nectin-4 is also considered an MeV host exit receptor [50].

### 3.3. CD46

In 1993, two groups [22,69] identified that human CD46 acts as a cellular receptor for laboratory adapted strains of MeV. Later on, though it was found that the wild type strain of MeV could not interact with CD46, it enlightened the mechanism of fusion activation in MeV and other Morbilliviruses [70,71]. On the contrary, CD46 could not act as a receptor for CDV and RPV [72,73]. CDV-H protein was found to interact with an unknown cellular receptor regulated by CD9 [74]. CD46 performs multiple functions, such as the modulation of T-cell functions [75], generation of regulatory T cells [76], and control of interferon (IFN) production [77]. Studies have indicated that MeV binds with CD46 at the Short Consensus Repeat 1 (SCR1) and Short Consensus Repeat 2 (SCR2) domains of the receptor [71,78,79,80]. Abundant studies on CD46 and MeV interactions have revealed that the region comprising CD46 residues 37–59 is involved in H-protein interactions [71], and mutation of R59 in the CCP1 domain (Complement Control Proteins) interferes with viral fusion and CD46 downregulation [81]. Interestingly, though CD46 is not a cell receptor for RPV, downregulation of CD46 was still observed in RPV, and in the case of PPRV only slight but significant downregulation of CD46 was noticed, whereas CDV was not found to cause any downregulation of CD46 [72]. This suggest that downregulation of CD46 is independent of its utilization as a receptor by Morbilliviruses.

### 3.4. Other Putative Receptors

Despite these well described receptors for PPRV, many factors have led us to think that there could be other unknown receptors involved in virus binding with the host. Manoharan et al. have proposed that sialic acid moieties could also be possible receptors for PPRV, as the virus hemagluttinates pig and chicken erythrocytes [82]. Likewise, Griffin has indicated that MeV not only infects immune cells, but also endothelial and neuronal cells which do not express SLAM [49]. This suggests that there could be other unknown receptors which interact with the virus. Recently, a study has indicated that haemagglutinin and hyperfusogenic proteins are involved in spreading the virus in a cell to cell manner between human neurons, without causing syncytium formation in the presence of some putative neuronal receptor for MeV [83]. Similarly, infection of neuronal and glial cells by PPRV has been reported where coinfection of PPRV with Border Disease virus has facilitated the passage of PPRV to the brain [84]. There have never been any reports of PPR virus alone infecting the brain. In the case of CDV, the neurovirulence, neurological disease phenotypes, and CNS areas targeted by the virus differ according to the CDV strains. However, the molecular determinants accounting for such differences are still unknown. It has been suggested that an unknown glial cell receptor known as “GliaR” is responsible for the persistence of CDV in the brain tissue from old dog encephalitis (ODE) affected dogs through a non-cytolytic astrocyte-to-astrocyte viral spread [63]. The C-type lectin DC specific intercellular adhesion molecule 3-grabbing non integrin DC-SIGN acts as an attachment receptor, enhancing CD46/CD150 mediated infection of dendritic cells in MeV [85]. Likewise, glycosaminoglycans (GAG) was also found to play role in infection through a tissue culture-adapted vaccine strain of RPV, recombinant with strains of CDV and MeV [86,87,88]. Melia et al. have demonstrated that Phocine Distemper virus (PDV) uses SLAM as a cell receptor, but does not utilize CD46 as a cell receptor [89]. Instead, PDV was found to use the proHB-EGF molecule as a receptor on Vero cells. Hence, in reference to these Morbillivirus receptors, there is a need to conduct more studies to investigate other putative receptors for the PPR virus.

## 4. Receptor Binding Domain (Ligand) on PPRV H Protein

Out of the six structural proteins of PPRV, the H and F proteins located on the surface of the viral particle are known to play a major role in binding to the host cell receptor, and in fusion between the viral envelope and the cell membrane. However, the basic role of H protein in progression of viral infection and specific binding to the host cell membrane is still not well-defined.

**Figure 1:**
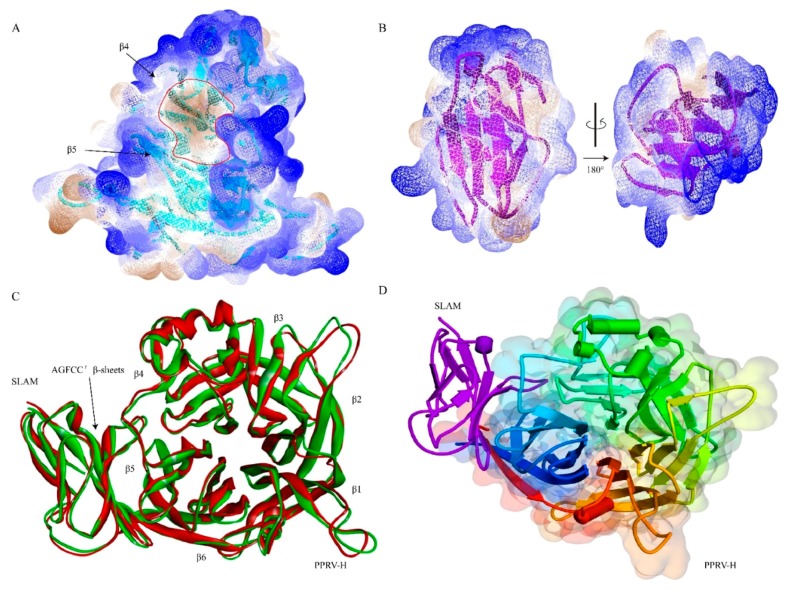
Cartoon and solvent drawing of the predicted PPRV H-SLAM complex. The complexes of the H protein’s six-bladed β-propeller head domain and the receptor SLAM V-like were built, in which β4–β6 of the head domain and the typical β-sandwich structure of the V domain were interacting surfaces. (**A** and **B**) show the analysis of the hydrophobicity of PPRV H and sheep SLAM, indicating that there was a strong hydrophobic groove between β4–β6 on the H head domain, and the surface on the sheep SLAM had stronger hydrophobicity. (**C**) A structure comparison revealed that the PPRV H-sheep SLAM and PPRV H-human SLAM complexes are very similar. (**D**) Interaction patterns between PPRV H and human SLAM.

The H protein is a disulphide-linked homo dimer consisting of an N-terminal cytoplasmic tail, a transmembrane region, a stalk, and a C-terminal receptor binding head domain. The N terminus is a signal peptide containing a hydrophobic domain that appears at the cytoplasmic membrane (amino acid position 35–38), whereas the C terminus protrudes on the outer side of the membrane, defining it as a type II glycoprotein [90]. Moreover, the head domain of the MeV H protein is the cubic shaped β propeller structure, which is different from the globular shape of the HN (haemagglutinin-neuraminidase) proteins of other Paramyxoviruses [91]. Additionally, the H protein is among the least conserved proteins of Morbilliviruses, sharing only 50% amino acid identity even with closely related viruses [92]. The binding sites of MeV H protein with cellular receptors have been extensively studied, and this has revealed that the β-sheet 4(β4) and β5 of the β propeller structure of the H protein interact with SCR1 and SCR2 of CD46 [93]. Subsequently, another study showed that the GFCC’C” region of the SLAM-V domain interacts with the β4-6 and loop regions on the lateral surface of the MeV-H β propeller structure [94]. On the other hand, a recent study on molecular evolution and characterization of PPRV H showed that the groove in the B4 blade and B5 of the head domain of PPRV H binds to the AGFCC’ β sheets of the membrane-distal ectodomain of sheep SLAM [95]. Furthermore, this study displayed C β-sheet, C’ β-sheet, and the loop of the two β sheets as the core regions of the interface. Our preliminary analysis of the hydrophobicity of PPRV H and sheep SLAM indicated that there was a strong hydrophobic groove between β4–β6 on the H head domain, and the surface on the sheep SLAM had stronger hydrophobicity. Additionally, structurally the PPRV H-sheep SLAM and PPRV H-human SLAM complexes are very similar (Figure 1). Interestingly, the H protein of PPRV agglutinates erythrocytes (hemagglutination activity), as well as cleaving sialic acid residue at the carbohydrate moiety in the glycoprotein of the host (neuraminidase activity) [96]. Hence, it is also called HN protein, as it performs both haemagglutinin and neuraminidase activities, whereas MeV-H protein lacks neuraminidase activity. In Morbilliviruses, the H protein mediates the initial attachment of the virus to a cell and induces fusion, promoting activity as a coexpression of the homotypic attachment. The fusion of the viral membrane and cellular membrane is achieved through the F protein, which is synthesized as the F0 precursor form in an inactive state. Later on, it is cleaved by the host machinery into transmembrane subunit F1 and surface subunit F2 fragments that are linked by a disulphide bond [97,98,99]. The F1 subunit possesses conserved domains, such as transmembrane (TM) domains, two heptad repeat regions A (HRA) and B (HRB), and an N-terminus hydrophobic fusion peptide [100,101]. F protein is highly conserved among Morbilliviruses, which may be the reason for cross protection among and between different genera of Morbilliviruses, as seen in the case of the RPV vaccine being used for immunizing against PPRV. Both the N-terminal domain and the C-terminal domain of this protein are associated with transferring proteins to the rough endoplasmic reticulum for translation and anchoring the protein in the membrane.

During the entry of the virus into the host cell, F protein attaches to the membrane and HRA (heptad repeat A) and HRB (heptad repeat B) interact with each other, resulting in fusion of the virus and host by bringing them close to each other [102,103]. PPRV F protein is known to have the capacity to cause cell-cell fusion independently, without HN protein, unlike in MeV, CDV and RPV where H protein is required for promoting fusion of the virus [104]. In the case of MeV, the binding of the virus with its receptors brings about conformational changes in the H protein, which are passed on to the F protein to trigger conformational changes which then expose its fusion peptide to become inserted into the cellular membrane [105,106,107]. Additionally, a recent study suggested that PPRV heptad region B (HRB) plays a more important role than heptad region A (HRA) in the viral fusion process [108]. On the other hand, Fengqi Xu (2018) showed the importance of interacting amino acid residues in binding to the receptors by the abinitio fragment molecular orbital (FMO) method [109]. According to this study, hydrophobic residues of MeV H have a strong tendency to interact with multiple receptors. In contrast, electrostatic interactions tend to interact with only specific molecular recognition. Hence, this method could also be used to study molecular interactions between the H protein and PPRV cellular receptors.

## 5. The Potential Role of Virus-Receptor Interaction in Host Range Restriction

Although goats and sheep are the major natural hosts of PPRV, an increasing number of domestic and wild animal species have been reported to be susceptible to PPRV over the last decades [110]. In recent years, PPRV has been reported to jump to other unusual hosts such as single humped camel, gazelle, ibex, gemsbok, deer, bushbuck, wild goats, and pigs [110]. These observations have raised concerns that PPRV may have the capacity to adapt to new hosts, which creates a challenge to attempts to eradicate PPR globally. Interestingly, it has been found that basal SLAM expression and PPRV replication are highly correlated. The expression of different levels of SLAM mRNA in different animals influences viral replication, thereby suggesting that SLAM mRNA expression levels could be one of the determinants of the different susceptibility of ruminant species to PPR. Notably, the study showed that the level of basal SLAM expression in peripheral blood mononuclear cells (PBMCs) of goats is highest compared to cattle, buffalo, and sheep [26].

One of the Morbilliviruses, CDV, which causes a fatal disease in domestic dogs, also affects wolves, foxes, raccoons, kinkajous, red pandas, black bears, giant pandas, ferrets, minks, civets, genets, spotted hyenas, lions, and tigers. In addition, there is evidence of CDV causing lethal infections in primates [111]. Further study demonstrated that the CDV monkey-adapted strain (CYN07-dV) was able to use human Nectin-4 for virus entry in vitro, and easily adapted to use human CD150 following minimal amino acid changes of the viral H protein [112]. Hence, as both SLAM and Nectin-4 of monkeys and human show high homology in their amino acid sequences [113], there is a significant potential threat of CDV occurring in human. Studies have also shown the spread of MeV in monkeys, causing a measles-like illness [114,115,116,117,118,119]. These findings indicate that change/adaptation of receptor binding specificity could play a crucial role in interspecies transmission. SLAM has been established as the principal host receptor for wild-type PPRV. Our preliminary study showed that the PPRV H-sheep SLAM and PPRV H-human SLAM complexes are very similar (unpublished data 2018, Figure 1). On the other hand, the mechanism by which PPRV uses the receptor to infect SLAM+ lymphocytes or Nectin-4+ epithelial cells are not completely clear. Recent progress identifying the crystal structure of the MeV-H protein and the MeV H-SLAM complex could help us to understand the role of the host-specific receptor binding properties in changes to host range restriction.

## 6. The Impact of Virus-Receptor Interactions on the Immune Response against PPRV

### 6.1. Innate Immune Response

The toll like receptor (TLR) plays a major role in innate immunity and also leads to the activation of apoptosis, phagocytosis, and complement and pro-inflammatory mediators, especially for the initiation of T-cell mediated immunity [120,121]. In addition, other pattern recognition receptors such as retinoic acid-inducible gene-1 (RIG-1) like receptor, melanoma differentiation antigen-5 (MDA-5) and nod like receptors (NLRs) [122] equally play a role in producing cytokines to produce an antiviral state [123,124]. PPRV-infected cells are responsible for the initiation of inflammation. TNFα produced by PPRV infection induces the stimulation of several immune cells that are able to induce fever, apoptosis, inflammation, and sepsis [125]. The interaction of MeV H protein with TLR2 stimulates interleukin 6 (IL-6) production and the expression of SLAM/CD150 on the cell surface, which causes immune activation and is effective against viral spread [126]. Neutralizing antibodies, the complement system, and cytokines are produced as an intrinsic mechanism to suppress viral replication. Infected lingual and buccal mucosa and lung epithelial tissue produce significantly increased inducible nitric oxide synthase (iNOS), IFN-γ and tumor necrosis factor (TNF-α) expression, which could play important role in the initiation and regulation of cytokine responses [127]. PPRV infection leads to the expression of cytokines such as IFNβ, IFNγ, IL-4, IL-1β, IL-8, IL-10, IL-6, and IL-12 [127,128]. INFs are potent cytokines produced in antiviral responses which can inhibit viral replication and have both immunostimulatory and immunomodulatory effects [129]. The RNA levels of IFN-β and IFN-γ increase during early infection, and decrease by day six [128]. However, there is no change in the levels of other pro-inflammatory cytokines (IL-1β, IL-6, and IL-8). According to the study by Atmaca and Kul [127], the epithelial lining of the oral cavity, lung, and tongue induce production of IFNγ in PPRV infected animals. Other organs, such as the lungs (bronchial epithelial cells) and lingual and buccal mucosa are also potent inducers of IFN-γ. A recent study showed dysregulation of 146 genes by PPRV internalization within 1 h post infection (hpi), of which 85 genes were upregulated and 61 genes were downregulated. This study further reported the association of NFKB1A, JUNB, and IL1A with PPRV infection in goats, and the generation of early cellular gene expression in caprine endometrial epithelial cells [130].

Viruses overcome the defense strategies of the host body through many mechanisms. In case of MeV, the C terminal domain of the V protein plays a significant role in inhibiting the IFN response by interacting with MDA5 [131,132]. Likewise, the binding of the P and V proteins with STAT 1 results in inhibition of phosphorylation of STAT1 and the signal cascade downstream to the IFNAR [133,134]. Similarly, PPRV V protein interferes with the IFN-I signaling pathway through its interaction with STAT 1 and STAT 2 to overcome the innate immune response [135]. PPRV V protein can more effectively inhibit the interferon stimulating responsive element (ISRE) and gamma-interferon-activation site (GAS) promoter expression than N and P proteins [135]. Morbilliviruses utilize different routes to block IFN-I and II signaling through the V protein. The V protein can impair signal transduction by binding with the Jak1/Tyk2 complex at the IFNAR receptor. However, V proteins of MeV, CDV, RPV, and PPRV can inhibit Tyk2 phosphorylation, whereas only RPV can inhibit Jak 1 phosphophorylation [136].

### 6.2. Adaptive Immune Response

As a response to a pathogen in the body, helper T cells are activated and secrete cytokines, which stimulate the proliferation and differentiation of the T cells themselves and activate other cells, including B cells, macrophages, and other lymphocytes. B cells then produce antibodies to overcome pathogen infection. Some cells (T and B cells) also differentiate into memory T cells so that the host can induce a fast immune response on reintroduction of the pathogen [137]. During Morbillivirus infection, including PPRV, a protective cell-mediated and humoral response is directed mainly against the H, F and N proteins [138,139,140], and lymphocytes play a major role in these immune responses. The H and F protein of PPRV were found to elicit PPRV specific B and T cell responses, along with neutralizing antibodies [44], whereas neutralizing antibodies were not detected against N proteins [141]. Moreover, antibody-dependent cell mediated cytotoxicity (ADCC) responses were found to play a role in immunity by recognizing PPRV F and H proteins, suggesting the role of ADCC and NK cells in modulating the course of PPRV infections [142]. The study further implied that PPRV infected ovine cells are the targets of the ADCC response.

Among PPRV proteins, although the N protein is the most abundant and more immunogenic due to being located closer to the promoter region, protective immunity is not induced by the N protein against the virus [92]. Through characterization of T cell epitopes in the PPRV N protein, it was found that N proteins of both PPRV and RPV induce a class I restricted, antigen specific and cross-reactive strong CD8+ T cell response [143]. CD8+ T cells recognize non-structural proteins (C or V) in PPRV and have a protective role in early infection by blocking the replication of the virus through the secretion of cytokines (IFN-γ) or MHC-linked cytotoxicity from these cells. Moreover, the presence of the T helper epitope at the amino-terminus and the linear B epitope at the carboxyl-terminus region in the 19 mer peptide in the N protein could help to induce antibodies with greater specificity. On the other hand, the antibodies produced against the glycoproteins H and F of RPV were shown to give protection against PPRV, although virus neutralizing antibodies were produced only against RPV [144]. A previous study on the recombinant vaccinia virus expressing the H or F of RPV was not found to produce neutralizing antibodies against PPRV, despite giving protection against PPRV infections [145]. However, the recombinant adenovirus expressing PPRV H or F proteins elicited protective B and T cell responses, along with neutralizing antibodies. This study further documented that the adeno-vectored vaccine is able to overcome PPRV-induced immunosuppression, and implied that adenovirus expressing PPRV proteins could be used to differentiate between vaccinated and infected animals [44]. Moreover, although there are efforts to establish reverse genetic-based recombinant vaccines [146,147] which could potentially differentiate between infected and vaccinated animals, this technology is still immature in PPRV and is not yet available to many researchers of PPRV [148]. Some studies have mapped at least four B cell epitopes using a set of monoclonal antibodies [149,150]. However, despite several research papers directed toward the role of B and T cell epitopes in the immunogenic proteins of PPRV, we are still lacking understanding of the molecular mechanism involved in the immune response, and the specific antibodies against PPRV still needed to be explored.

### 6.3. Inhibitory Impact of Viral Protein-SLAM Interactions on the Immune Response

Morbilliviruses are highly immunosuppressive in nature, providing an opportunity for secondary bacterial infections. On the other hand, strong immune responses are generated against Morbilliviruses after infection, resulting in long term immunity [151,152]. The infection of peripheral blood lymphocytes and lymphoid tissues by Morbilliviruses leads to the inhibition of IFN production, suppression of the inflammatory response, inhibition of immunoglobulin synthesis (due to loss of B cells), or cell cycle arrest [153]. The mechanism of immunosuppression in PPRV and in other Morbilliviruses is still poorly understood. A previous study on MeV revealed that surface contact between one or more viral proteins (H and F), rather than soluble factors, induces proliferative inhibition of peripheral blood lymphocytes (PBL) and further indicated that the infection of a small number of PBLs by MeV can induce a general unresponsiveness of uninfected PBLs by surface contact, thereby resulting in general suppression of the immune response [154]. Similarly, Heaney and colleagues demonstrated that co-expression of both the F and H protein of RPV induces immunosuppression, as in MeV [155]. In addition, the study showed that all Morbilliviruses, including PPRV, inhibit lymphoid cell proliferation, and PPRV was found to induce a stronger immunosuppressive effect on caprine peripheral blood lymphocytes (50%) compared to RPV (30%) [155]. Besides H and F, the other viral proteins, N and P, also play an important role in inhibition of lymphoproliferation and induction of lymphodepletion in Morbilliviruses (MeV and RPV) [154,155,156,157,158]. The N protein impairs antibody production by binding to Fc-γ RII on human and murine B cells [159], and the glycoprotein complex impairs T cell proliferation [160,161]. Similarly, the N protein of PPRV has been shown to suppress lymphoproliferation, resulting in immunosuppression [162]. Furthermore, the same study showed that the amino acid sequence 1–420 of the truncated nucleoprotein of PPRV is responsible for inducing immunosuppression, like in other Morbilliviruses [162]. Dendritic cells (DCs) and lymphocytes expressing SLAM/CD150 receptor are the major targets of MeV infection, thereby interfering with the normal functions of CD150 in the host body [163]. Furthermore, the interaction of MeV and CD150 leads to the downregulation of receptor expression, resulting in immunosuppression [164,165]. Romanets-Korbut and colleagues in 2016 indicated that MeV H interaction with CD150 increases Akt phosphorylation in human dendritic cells (DCs) during early infection and decreases p38 MAPK phosphorylation in DCs, thereby modulating the signal transduction pathway and inhibiting the host immune response [166]. This study has further shown that the interaction between MeV H and human DCs could lead to inhibition of IL-12 production, resulting in immunosuppression during MeV infection.

Non-structural proteins of Morbilliviruses play an important role in antagonizing the immune response. A previous study has shown that PPRV V protein actively inhibits IFN-β induction by binding to MDA-5 and, to a lesser extent, RIG-I; it is not the sole viral protein for blocking IFN-β induction [167]. Hahm et al. demonstrated that the Measles virus V and C proteins are not responsible for IL-12 suppression mediated via TLR 4 signaling; instead, MeV H binding to human SLAM facilitates TLR-4 mediated IL-12 suppression [168]. The V protein of MeV inhibits IFN-α/β and NF-кB (nuclear factor kappa B) signaling [169], whereas in RPV, the C protein impairs the induction of type I IFN [170] and the P protein interacts with STAT 1 and IFN signaling.

## 7. Conclusions

PPR is one of the priority animal diseases whose control is essential for poverty alleviation. The interaction of the virus with host cell receptors determines virus tropism and tissue susceptibility. In this review, we discussed the PPR virus, recent insights into cellular receptors and other potential receptors, the host immune response towards PPRV infection, and the probable mechanisms behind immunosuppression due to PPRV infection. This review also attempted to provide valuable information on different cellular receptors used by other Morbilliviruses, which could be explored for PPRV. Most of the information regarding PPRV immune responses and immunosuppression has been presumed based on comparison with MeV, which has been studied extensively. Therefore, further investigation is highly recommended to provide a clearer and more comprehensive understanding of PPRV interactions with host cellular receptors and the mechanism of the host immune response, which will contribute to devising advanced and effective control strategies.

## References

[1] OIE Geographical Distribution of PPR. http://www.oie.int/en/animal-health-in-the-world/ppr-portal/distribution/.

[2] Nanda Y.P., Chatterjee A., Purohit A.K., Diallo A., Innui K., Sharma R.N., Libeau G., Thevasagayam J.A., Bruning A., Kitching R.P. (1996). The isolation of peste des petits ruminants virus from northern India. Vet. Microbiol..

[3] Truong T., Boshra H., Embury-Hyatt C., Nfon C., Gerdts V., Tikoo S., Babiuk L.A., Kara P., Chetty T., Mather A. (2014). Peste des petits ruminants virus tissue tropism and pathogenesis in sheep and goats following experimental infection. PLoS ONE.

[4] Parida S., Muniraju M., Altan E., Baazizi R., Raj G.D., Mahapatra M. (2016). Emergence of PPR and its threat to Europe. Small Rumin. Res..

[5] Khan H.A., Siddique M., Abubakar M., Ashraf M. (2008). The detection of antibody against peste des petits ruminants virus in sheep, goats, cattle and buffaloes. Trop. Anim. Health Prod..

[6] Pope R.A., Parida S., Bailey D., Brownlie J., Barrett T., Banyard A.C. (2013). Early events following experimental infection with Peste-Des-Petits ruminants virus suggest immune cell targeting. PLoS ONE.

[7] Scott G.R., Gibbs E.P.J. (1981). Rinderpest and peste des petits ruminants. Virus Diseases of Food Animals. A World Geography of Epidemiology and Control.

[8] Kahn C.M. (2008). Peste Des Petits Ruminants. The Merck Veterinary Manual.

[9] Barrett T., Amarel-Doel C., Kitching R.P., Gusev A. (1993). Use of the polymerase chain reaction in differentiating rinderpest field virus and vaccine virus in the same animals. Rev. Sci. Tech..

[10] Bailey D., Banyard A., Dash P., Ozkul A., Barrett T. (2005). Full genome sequence of peste des petits ruminants virus, a member of the Morbillivirus genus. Virus Res..

[11] Durojaiye O.A., Taylor W.P., Smale C. (1985). The ultrastructure of peste des petits ruminants virus. Zent. Fur Vet. Reihe B. J. Vet. Med. Ser. B.

[12] Gibbs E.P., Taylor W.P., Lawman M.J., Bryant J. (1979). Classification of peste des petits ruminants virus as the fourth member of the genus Morbillivirus. Intervirology.

[13] Diallo A. (1990). Morbillivirus group: Genome organisation and proteins. Vet. Microbiol..

[14] Crowley J.C., Dowling P.C., Menonna J., Silverman J.I., Schuback D., Cook S.D., Blumberg B.M. (1988). Sequence variability and function of measles virus 3′ and 5′ ends and intercistronic regions. Virology.

[15] Diallo A., Barrett T., Barbron M., Meyer G., Lefevre P.C. (1994). Cloning of the nucleocapsid protein gene of peste-des-petits-ruminants virus: Relationship to other morbilliviruses. J. Gen. Virol..

[16] Haas L., Baron M.D., Liess B., Barrett T. (1995). Editing of morbillivirus P gene transcripts in infected animals. Vet. Microbiol..

[17] Sidhu M.S., Husar W., Cook S.D., Dowling P.C., Udem S.A. (1993). Canine distemper terminal and intergenic non-protein coding nucleotide sequences: Completion of the entire CDV genome sequence. Virology.

[18] Lamb R.A., Kolakofsky D., Fields B.N., Knipe D.N., Howley P.M. (2001). Paramyxoviridae: The viruses and their replication. Fields Virology.

[19] Dhar P., Sreenivasa B.P., Barrett T., Corteyn M., Singh R.P., Bandyopadhyay S.K. (2002). Recent epidemiology of peste des petits ruminants virus (PPRV). Vet. Microbiol..

[20] Birch J., Juleff N., Heaton M.P., Kalbfleisch T., Kijas J., Bailey D. (2013). Characterization of ovine Nectin-4, a novel peste des petits ruminants virus receptor. J. Virol..

[21] Pawar R.M., Raj G.D., Kumar T.M.A.S., Raja A., Balachandran C. (2008). Effect of siRNA mediated suppression of signaling lymphocyte activation molecule on replication of peste des petits ruminants virus in vitro. Virus Res..

[22] Dorig R.E., Marcil A., Chopra A., Richardson C.D. (1993). The human CD46 molecule is a receptor for measles virus (Edmonston strain). Cell.

[23] Yang B., Qi X., Guo H., Jia P., Chen S., Chen Z., Wang T., Wang J., Xue Q. (2018). Peste des Petits Ruminants Virus Enters Caprine Endometrial Epithelial Cells via the Caveolae-Mediated Endocytosis Pathway.

[24] Tatsuo H., Ono N., Yanagi Y. (2001). Morbilliviruses use signaling lymphocyte activation molecules (CD150) as cellular receptors. J. Virol..

[25] Tatsuo H., Ono N., Tanaka K., Yanagi Y. (2000). SLAM (CDw150) is a cellular receptor for measles virus. Nature.

[26] Pawar R.M., Dhinakar Raj G., Balachandran C. (2008). Relationship between the level of signaling lymphocyte activation molecule mRNA and replication of Peste-des-petits-ruminants virus in peripheral blood mononuclear cells of host animals. Acta Virol..

[27] Sarkar J., Balamurugan V., Sen A., Saravanan P., Sahay B., Rajak K.K., Rasool T.J., Bhanuprakash V., Singh R.K. (2009). Sequence analysis of morbillivirus CD150 receptor-Signaling Lymphocyte Activation Molecule (SLAM) of different animal species. Virus Genes.

[28] Ohishi K., Ando A., Suzuki R., Takishita K., Kawato M., Katsumata E., Ohtsu D., Okutsu K., Tokutake K., Miyahara H. (2010). Host–virus specificity of morbilliviruses predicted by structural modeling of the marine mammal SLAM, a receptor. Comp. Immunol. Microbiol. Infect. Dis..

[29] Veillette A., Latour S. (2003). The SLAM family of immune-cell receptors. Curr. Opin. Immunol..

[30] Vincent S., Spehner D., Manie S., Delorme R., Drillien R., Gerlier D. (1999). Inefficient measles virus budding in murine L.CD46 fibroblasts. Virology.

[31] Schwartzberg P.L., Mueller K.L., Qi H., Cannons J.L. (2009). SLAM receptors and SAP influence lymphocyte interactions, development and function. Nat. Rev. Immunol..

[32] Cocks B.G., Chang C.C., Carballido J.M., Yssel H., de Vries J.E., Aversa G. (1995). A novel receptor involved in T-cell activation. Nature.

[33] Tangye S.G., Phillips J.H., Lanier L.L. (2000). The CD2-subset of the Ig superfamily of cell surface molecules: Receptor-ligand pairs expressed by NK cells and other immune cells. Semin. Immunol..

[34] Ono N., Tatsuo H., Tanaka K., Minagawa H., Yanagi Y. (2001). V domain of human SLAM (CDw150) is essential for its function as a measles virus receptor. J. Virol..

[35] Ohno S., Seki F., Ono N., Yanagi Y. (2003). Histidine at position 61 and its adjacent amino acid residues are critical for the ability of SLAM (CD150) to act as a cellular receptor for measles virus. J. Gen. Virol..

[36] Castro A.G., Hauser T.M., Cocks B.G., Abrams J., Zurawski S., Churakova T., Zonin F., Robinson D., Tangye S.G., Aversa G. (1999). Molecular and functional characterization of mouse signaling lymphocytic activation molecule (SLAM): Differential expression and responsiveness in Th1 and Th2 cells. J. Immunol..

[37] Ono N., Tatsuo H., Hidaka Y., Aoki T., Minagawa H., Yanagi Y. (2001). Measles viruses on throat swabs from measles patients use signaling lymphocytic activation molecule (CDw150) but not CD46 as a cellular receptor. J. Virol..

[38] Yanagi Y., Takeda M., Ohno S., Hashiguchi T. (2009). Measles virus receptors. Curr. Top. Microbiol. Immunol..

[39] Lu G., Gao G.F., Yan J. (2013). The receptors and entry of measles virus: A review. Sheng Wu Gong Cheng Xue Bao.

[40] Navaratnarajah C.K., Vongpunsawad S., Oezguen N., Stehle T., Braun W., Hashiguchi T., Maenaka K., Yanagi Y., Cattaneo R. (2008). Dynamic interaction of the measles virus hemagglutinin with its receptor signaling lymphocytic activation molecule (SLAM, CD150). J. Biol. Chem..

[41] De Vries R.D., McQuaid S., van Amerongen G., Yuksel S., Verburgh R.J., Osterhaus A.D., Duprex W.P., de Swart R.L. (2012). Measles immune suppression: Lessons from the macaque model. PLoS Pathog..

[42] Laksono B.M., Grosserichter-Wagener C., de Vries R.D., Langeveld S.A.G., Brem M.D., van Dongen J.J.M., Katsikis P.D., Koopmans M.P.G., van Zelm M.C., de Swart R.L. (2018). In Vitro Measles Virus Infection of Human Lymphocyte Subsets Demonstrates High Susceptibility and Permissiveness of both Naive and Memory B Cells. J. Virol..

[43] Condack C., Grivel J.C., Devaux P., Margolis L., Cattaneo R. (2007). Measles virus vaccine attenuation: Suboptimal infection of lymphatic tissue and tropism alteration. J. Infect. Dis..

[44] Rojas J.M., Moreno H., Valcarcel F., Pena L., Sevilla N., Martin V. (2014). Vaccination with recombinant adenoviruses expressing the peste des petits ruminants virus F or H proteins overcomes viral immunosuppression and induces protective immunity against PPRV challenge in sheep. PLoS ONE.

[45] Herbert R., Baron J., Batten C., Baron M., Taylor G. (2014). Recombinant adenovirus expressing the haemagglutinin of Peste des petits ruminants virus (PPRV) protects goats against challenge with pathogenic virus; a DIVA vaccine for PPR. Vet. Res..

[46] Manjunath S., Kumar G.R., Mishra B.P., Mishra B., Sahoo A.P., Joshi C.G., Tiwari A.K., Rajak K.K., Janga S.C. (2015). Genomic analysis of host—Peste des petits ruminants vaccine viral transcriptome uncovers transcription factors modulating immune regulatory pathways. Vet. Res..

[47] Mondal B., Sreenivasa B.P., Dhar P., Singh R.P., Bandyopadhyay S.K. (2001). Apoptosis induced by peste des petits ruminants virus in goat peripheral blood mononuclear cells. Virus Res..

[48] Kul O., Kabakci N., Atmaca H.T., Ozkul A. (2007). Natural peste des petits ruminants virus infection: Novel pathologic findings resembling other morbillivirus infections. Vet Pathol..

[49] Griffin D.E., Knipe D.M., Howley P.M., Griffin D.E., Lamb R.A., Martin M.A., Roizman B., Straus S.E. (2007). Measles virus. Fields Virology.

[50] Muhlebach M.D., Mateo M., Sinn P.L., Prufer S., Uhlig K.M., Leonard V.H., Navaratnarajah C.K., Frenzke M., Wong X.X., Sawatsky B. (2011). Adherens junction protein nectin-4 is the epithelial receptor for measles virus. Nature.

[51] Noyce R.S., Bondre D.G., Ha M.N., Lin L.T., Sisson G., Tsao M.S., Richardson C.D. (2011). Tumor cell marker PVRL4 (nectin 4) is an epithelial cell receptor for measles virus. PLoS Pathog..

[52] Delpeut S., Noyce R.S., Richardson C.D. (2014). The tumor-associated marker, PVRL4 (nectin-4), is the epithelial receptor for morbilliviruses. Viruses.

[53] Delpeut S., Noyce R.S., Richardson C.D. (2014). The V domain of dog PVRL4 (nectin-4) mediates canine distemper virus entry and virus cell-to-cell spread. Virology.

[54] Takai Y., Miyoshi J., Ikeda W., Ogita H. (2008). Nectins and nectin-like molecules: Roles in contact inhibition of cell movement and proliferation. Nat. Rev. Mol. Cell Biol..

[55] Fabre S., Reymond N., Cocchi F., Menotti L., Dubreuil P., Campadelli-Fiume G., Lopez M. (2002). Prominent role of the Ig-like V domain in trans-interactions of nectins. Nectin3 and nectin 4 bind to the predicted C-C′-C″-D beta-strands of the nectin1 V domain. J. Biol. Chem..

[56] Takai Y., Ikeda W., Ogita H., Rikitake Y. (2008). The immunoglobulin-like cell adhesion molecule nectin and its associated protein afadin. Annu. Rev. Cell Dev. Biol..

[57] Satoh-Horikawa K., Nakanishi H., Takahashi K., Miyahara M., Nishimura M., Tachibana K., Mizoguchi A., Takai Y. (2000). Nectin-3, a new member of immunoglobulin-like cell adhesion molecules that shows homophilic and heterophilic cell-cell adhesion activities. J. Biol. Chem..

[58] Mendelsohn C.L., Wimmer E., Racaniello V.R. (1989). Cellular receptor for poliovirus: Molecular cloning, nucleotide sequence, and expression of a new member of the immunoglobulin superfamily. Cell.

[59] Takahashi K., Nakanishi H., Miyahara M., Mandai K., Satoh K., Satoh A., Nishioka H., Aoki J., Nomoto A., Mizoguchi A. (1999). Nectin/PRR: An immunoglobulin-like cell adhesion molecule recruited to cadherin-based adherens junctions through interaction with Afadin, a PDZ domain-containing protein. J. Cell Biol..

[60] Reymond N., Fabre S., Lecocq E., Adelaide J., Dubreuil P., Lopez M. (2001). Nectin4/PRR4, a new afadin-associated member of the nectin family that trans-interacts with nectin1/PRR1 through V domain interaction. J. Biol. Chem..

[61] Pratakpiriya W., Seki F., Otsuki N., Sakai K., Fukuhara H., Katamoto H., Hirai T., Maenaka K., Techangamsuwan S., Lan N.T. (2012). Nectin4 is an epithelial cell receptor for canine distemper virus and involved in neurovirulence. J. Virol..

[62] Pratakpiriya W., Ping Teh A.P., Radtanakatikanon A., Pirarat N., Thi Lan N., Takeda M., Techangamsuwan S., Yamaguchi R. (2017). Expression of canine distemper virus receptor nectin-4 in the central nervous system of dogs. Sci. Rep..

[63] Alves L., Khosravi M., Avila M., Ader-Ebert N., Bringolf F., Zurbriggen A., Vandevelde M., Plattet P. (2015). SLAM- and Nectin-4-Independent Noncytolytic Spread of Canine Distemper Virus in Astrocytes. J. Virol..

[64] Fakri F., Elarkam A., Daouam S., Tadlaoui K., Fassi-Fihri O., Richardson C.D., Elharrak M. (2016). VeroNectin-4 is a highly sensitive cell line that can be used for the isolation and titration of Peste des Petits Ruminants virus. J. Virol. Methods.

[65] Ogita H., Rikitake Y., Miyoshi J., Takai Y. (2010). Cell adhesion molecules nectins and associating proteins: Implications for physiology and pathology. Proc. Jpn. Acad. Ser. B Phys. Biol. Sci..

[66] Abdullah H., Brankin B., Brady C., Cosby S.L. (2013). Wild-type measles virus infection upregulates poliovirus receptor-related 4 and causes apoptosis in brain endothelial cells by induction of tumor necrosis factor-related apoptosis-inducing ligand. J. Neuropathol. Exp Neurol..

[67] Leonard V.H., Sinn P.L., Hodge G., Miest T., Devaux P., Oezguen N., Braun W., McCray P.B., McChesney M.B., Cattaneo R. (2008). Measles virus blind to its epithelial cell receptor remains virulent in rhesus monkeys but cannot cross the airway epithelium and is not shed. J. Clin. Investig..

[68] Ludlow M., Rennick L.J., Sarlang S., Skibinski G., McQuaid S., Moore T., de Swart R.L., Duprex W.P. (2010). Wild-type measles virus infection of primary epithelial cells occurs via the basolateral surface without syncytium formation or release of infectious virus. J. Gen. Virol..

[69] Naniche D., Varior-Krishnan G., Cervoni F., Wild T.F., Rossi B., Rabourdin-Combe C., Gerlier D. (1993). Human membrane cofactor protein (CD46) acts as a cellular receptor for measles virus. J. Virol..

[70] Buchholz C.J., Schneider U., Devaux P., Gerlier D., Cattaneo R. (1996). Cell entry by measles virus: Long hybrid receptors uncouple binding from membrane fusion. J. Virol..

[71] Buchholz C.J., Koller D., Devaux P., Mumenthaler C., Schneider-Schaulies J., Braun W., Gerlier D., Cattaneo R. (1997). Mapping of the primary binding site of measles virus to its receptor CD46. J. Biol. Chem..

[72] Galbraith S.E., Tiwari A., Baron M.D., Lund B.T., Barrett T., Cosby S.L. (1998). Morbillivirus downregulation of CD46. J. Virol..

[73] Naniche D., Wild T.F., Rabourdin-Combe C., Gerlier D. (1993). Measles virus haemagglutinin induces down-regulation of gp57/67, a molecule involved in virus binding. J. Gen. Virol..

[74] Singethan K., Topfstedt E., Schubert S., Duprex W.P., Rima B.K., Schneider-Schaulies J. (2006). CD9-dependent regulation of Canine distemper virus-induced cell-cell fusion segregates with the extracellular domain of the haemagglutinin. J. Gen. Virol..

[75] Marie J.C., Astier A.L., Rivailler P., Rabourdin-Combe C., Wild T.F., Horvat B. (2002). Linking innate and acquired immunity: Divergent role of CD46 cytoplasmic domains in T cell–induced inflammation. Nature Immunol..

[76] Kemper C., Chan A.C., Green J.M., Brett K.A., Murphy K.M., Atkinson J.P. (2003). Activation of human CD4+ cells with CD3 and CD46 induces a T-regulatory cell 1 phenotype. Nature.

[77] Katayama Y., Hirano A., Wong T.C. (2000). Human Receptor for Measles Virus (CD46) Enhances Nitric Oxide Production and Restricts Virus Replication in Mouse Macrophages by Modulating Production of Alpha/Beta Interferon. J. Virol..

[78] Casasnovas J.M., Larvie M., Stehle T. (1999). Crystal structure of two CD46 domains reveals an extended measles virus-binding surface. EMBO J..

[79] Christiansen D., Deleage G., Gerlier D. (2000). Evidence for distinct complement regulatory and measles virus binding sites on CD46 SCR2. Eur. J. Immunol..

[80] Hsu E.C., Dörig R.E., Sarangi F., Marcil A., Iorio C., Richardson C.D. (1997). Artificial mutations and natural variations in the CD46 molecules from human and monkey cells define regions important for measles virus binding. J. Virol..

[81] Hsu E.C., Sabatinos S., Hoedemaeker F.J., Rose D.R., Richardson C.D. (1999). Use of site-specific mutagenesis and monoclonal antibodies to map regions of CD46 that interact with measles virus H protein. Virology.

[82] Manoharan S., Jayakumar R., Govindarajan R., Koteeswaran A. (2005). Haemagglutination as a confirmatory test for Peste des petits ruminants diagnosis. Small Rumin. Res..

[83] Sato Y., Watanabe S., Fukuda Y., Hashiguchi T., Yanagi Y., Ohno S. (2018). Cell-to-Cell Measles Virus Spread between Human Neurons Is Dependent on Hemagglutinin and Hyperfusogenic Fusion Protein. J. Virol..

[84] Toplu N., Oguzoglu T.C., Albayrak H. (2012). Dual infection of fetal and neonatal small ruminants with border disease virus and peste des petits ruminants virus (PPRV): Neuronal tropism of PPRV as a novel finding. J. Comp. Pathol..

[85] De Witte L., de Vries R.D., van der Vlist M., Yuksel S., Litjens M., de Swart R.L., Geijtenbeek T.B. (2008). DC-SIGN and CD150 have distinct roles in transmission of measles virus from dendritic cells to T-lymphocytes. PLoS Pathog..

[86] Fujita K., Miura R., Yoneda M., Shimizu F., Sato H., Muto Y., Endo Y., Tsukiyama-Kohara K., Kai C. (2007). Host range and receptor utilization of canine distemper virus analyzed by recombinant viruses: Involvement of heparin-like molecule in CDV infection. Virology.

[87] Terao-Muto Y., Yoneda M., Seki T., Watanabe A., Tsukiyama-Kohara K., Fujita K., Kai C. (2008). Heparin-like glycosaminoglycans prevent the infection of measles virus in SLAM-negative cell lines. Antivir. Res..

[88] Baron M.D. (2005). Wild-type Rinderpest virus uses SLAM (CD150) as its receptor. J. Gen. Virol..

[89] Melia M.M., Earle J.P., Abdullah H., Reaney K., Tangy F., Cosby S.L. (2014). Use of SLAM and PVRL4 and identification of pro-HB-EGF as cell entry receptors for wild type phocine distemper virus. PLoS ONE.

[90] Vongpunsawad S., Oezgun N., Braun W., Cattaneo R. (2004). Selectively receptor-blind measles viruses: Identification of residues necessary for SLAM- or CD46-induced fusion and their localization on a new hemagglutinin structural model. J. Virol..

[91] Hashiguchi T., Kajikawa M., Maita N., Takeda M., Kuroki K., Sasaki K., Kohda D., Yanagi Y., Maenaka K. (2007). Crystal structure of measles virus hemagglutinin provides insight into effective vaccines. Proc. Natl. Acad. Sci. USA.

[92] Munir M., Zohari S., Berg M. (2013). Molecular Biology and Pathogenesis of Peste des Petits Ruminants Virus. Springer Briefs in Animal Sciences.

[93] Santiago C., Celma M.L., Stehle T., Casasnovas J.M. (2010). Structure of the measles virus hemagglutinin bound to the CD46 receptor. Nat. Struct. Mol. Biol..

[94] Hashiguchi T., Ose T., Kubota M., Maita N., Kamishikiryo J., Maenaka K., Yanagi Y. (2011). Structure of the measles virus hemagglutinin bound to its cellular receptor SLAM. Nat. Struct. Mol. Biol..

[95] Liang Z., Yuan R., Chen L., Zhu X., Dou Y. (2016). Molecular Evolution and Characterization of Hemagglutinin (H) in Peste des Petits Ruminants Virus. PLoS ONE.

[96] Langedijk J.P., Daus F.J., van Oirschot J.T. (1997). Sequence and structure alignment of Paramyxoviridae attachment proteins and discovery of enzymatic activity for a morbillivirus hemagglutinin. J. Virol..

[97] Scheid A., Choppin P.W. (1974). Identification of biological activities of paramyxovirus glycoproteins. Activation of cell fusion, hemolysis, and infectivity of proteolytic cleavage of an inactive precursor protein of Sendai virus. Virology.

[98] Morrison T., Ward L.J., Semerjian A. (1985). Intracellular processing of the Newcastle disease virus fusion glycoprotein. J. Virol..

[99] Paal T., Brindley M.A., St Clair C., Prussia A., Gaus D., Krumm S.A., Snyder J.P., Plemper R.K. (2009). Probing the spatial organization of measles virus fusion complexes. J. Virol..

[100] Rahaman A., Srinivasan N., Shamala N., Shaila M.S. (2003). The fusion core complex of the peste des petits ruminants virus is a six-helix bundle assembly. Biochemistry.

[101] Yin H.S., Wen X., Paterson R.G., Lamb R.A., Jardetzky T.S. (2006). Structure of the parainfluenza virus 5 F protein in its metastable, prefusion conformation. Nature.

[102] Muhlebach M.D., Leonard V.H., Cattaneo R. (2008). The measles virus fusion protein transmembrane region modulates availability of an active glycoprotein complex and fusion efficiency. J. Virol..

[103] Lee J.K., Prussia A., Snyder J.P., Plemper R.K. (2007). Reversible inhibition of the fusion activity of measles virus F protein by an engineered intersubunit disulfide bridge. J. Virol..

[104] Seth S., Shaila M.S. (2001). The fusion protein of Peste des petits ruminants virus mediates biological fusion in the absence of hemagglutinin-neuraminidase protein. Virology.

[105] Plemper R.K., Brindley M.A., Iorio R.M. (2011). Structural and mechanistic studies of measles virus illuminate paramyxovirus entry. PLoS Pathog..

[106] Schneider-Schaulies S., ter Meulen V. (2002). Measles virus and immunomodulation: Molecular bases and perspectives. Expert Rev. Mol. Med..

[107] Yanagi Y., Takeda M., Ohno S. (2006). Measles virus: Cellular receptors, tropism and pathogenesis. J. Gen. Virol..

[108] Meng X., Deng R., Zhu X., Zhang Z. (2018). Quantitative investigation of the direct interaction between Hemagglutinin and fusion proteins of Peste des petits ruminant virus using surface Plasmon resonance. Virol. J..

[109] Xu F., Tanaka S., Watanabe H., Shimane Y., Iwasawa M., Ohishi K., Maruyama T. (2018). Computational Analysis of the Interaction Energies between Amino Acid Residues of the Measles Virus Hemagglutinin and Its Receptors. Viruses.

[110] Wohlsein P., Singh R.P., Munir M. (2015). Peste des petits ruminants in unusual hosts: Epidemiology, disease, and impact on eradication. Peste Des Petits Ruminants Virus.

[111] Beineke A., Baumgartner W., Wohlsein P. (2015). Cross-species transmission of canine distemper virus-an update. One Health.

[112] Sakai K., Yoshikawa T., Seki F., Fukushi S., Tahara M., Nagata N., Ami Y., Mizutani T., Kurane I., Yamaguchi R. (2013). Canine distemper virus associated with a lethal outbreak in monkeys can readily adapt to use human receptors. J. Virol..

[113] Sakai K., Nagata N., Ami Y., Seki F., Suzaki Y., Iwata-Yoshikawa N., Suzuki T., Fukushi S., Mizutani T., Yoshikawa T. (2013). Lethal canine distemper virus outbreak in cynomolgus monkeys in Japan in 2008. J. Virol..

[114] Auwaerter P.G., Rota P.A., Elkins W.R., Adams R.J., DeLozier T., Shi Y., Bellini W.J., Murphy B.R., Griffin D.E. (1999). Measles virus infection in rhesus macaques: Altered immune responses and comparison of the virulence of six different virus strains. J. Infect. Dis..

[115] Bankamp B., Hodge G., McChesney M.B., Bellini W.J., Rota P.A. (2008). Genetic changes that affect the virulence of measles virus in a rhesus macaque model. Virology.

[116] De Swart R.L. (2009). Measles studies in the macaque model. Curr. Top. Microbiol. Immunol..

[117] Kobune F., Takahashi H., Terao K., Ohkawa T., Ami Y., Suzaki Y., Nagata N., Sakata H., Yamanouchi K., Kai C. (1996). Nonhuman primate models of measles. Lab. Anim. Sci..

[118] McChesney M.B., Fujinami R.S., Lerche N.W., Marx P.A., Oldstone M.B. (1989). Virus-induced immunosuppression: Infection of peripheral blood mononuclear cells and suppression of immunoglobulin synthesis during natural measles virus infection of rhesus monkeys. J. Infect. Dis..

[119] McChesney M.B., Miller C.J., Rota P.A., Zhu Y.D., Antipa L., Lerche N.W., Ahmed R., Bellini W.J. (1997). Experimental measles. I. Pathogenesis in the normal and the immunized host. Virology.

[120] Janeway C.A., Medzhitov R. (2002). Innate immune recognition. Annu. Rev. Immunol..

[121] West N.P., Pyne D.B., Renshaw G., Cripps A.W. (2006). Antimicrobial peptides and proteins, exercise and innate mucosal immunity. Fems Immunol. Med. Microbiol..

[122] OIE Peste Des Petits Ruminants. http://www.oie.int/en/animal-health-in-the-world/official-disease-status/peste-des-petits-ruminants/.

[123] Randall R.E., Goodbourn S. (2008). Interferons and viruses: An interplay between induction, signalling, antiviral responses and virus countermeasures. J. Gen. Virol..

[124] Platanias L.C. (2005). Mechanisms of type-I- and type-II-interferon-mediated signalling. Nat. Rev. Immunol..

[125] Van Riel D., Leijten L.M., van der Eerden M., Hoogsteden H.C., Boven L.A., Lambrecht B.N., Osterhaus A.D., Kuiken T. (2011). Highly pathogenic avian influenza virus H5N1 infects alveolar macrophages without virus production or excessive TNF-alpha induction. PLoS Pathog..

[126] Bieback K., Lien E., Klagge I.M., Avota E., Schneider-Schaulies J., Duprex W.P., Wagner H., Kirschning C.J., Ter Meulen V., Schneider-Schaulies S. (2002). Hemagglutinin protein of wild-type measles virus activates toll-like receptor 2 signaling. J. Virol..

[127] Atmaca H.T., Kul O. (2012). Examination of epithelial tissue cytokine response to natural peste des petits ruminants virus (PPRV) infection in sheep and goats by immunohistochemistry. Histol. Histopathol..

[128] Baron J., Bin-Tarif A., Herbert R., Frost L., Taylor G., Baron M.D. (2014). Early changes in cytokine expression in peste des petits ruminants disease. Vet. Res..

[129] Koyama S., Ishii K.J., Coban C., Akira S. (2008). Innate immune response to viral infection. Cytokine.

[130] Yang B., Qi X., Chen Z., Chen S., Xue Q., Jia P., Wang T., Wang J. (2018). Binding and entry of peste des petits ruminants virus into caprine endometrial epithelial cells profoundly affect early cellular gene expression. Vet. Res..

[131] Andrejeva J., Childs K.S., Young D.F., Carlos T.S., Stock N., Goodbourn S., Randall R.E. (2004). The V proteins of paramyxoviruses bind the IFN-inducible RNA helicase, mda-5, and inhibit its activation of the IFN-beta promoter. Proc. Natl. Acad. Sci. USA.

[132] Childs K., Stock N., Ross C., Andrejeva J., Hilton L., Skinner M., Randall R., Goodbourn S. (2007). mda-5, but not RIG-I, is a common target for paramyxovirus V proteins. Virology.

[133] Caignard G., Guerbois M., Labernardiere J.L., Jacob Y., Jones L.M., Wild F., Tangy F., Vidalain P.O. (2007). Measles virus V protein blocks Jak1-mediated phosphorylation of STAT1 to escape IFN-alpha/beta signaling. Virology.

[134] Devaux P., von Messling V., Songsungthong W., Springfeld C., Cattaneo R. (2007). Tyrosine 110 in the measles virus phosphoprotein is required to block STAT1 phosphorylation. Virology.

[135] Ma X., Yang X., Nian X., Zhang Z., Dou Y., Zhang X., Luo X., Su J., Zhu Q., Cai X. (2015). Identification of amino-acid residues in the V protein of peste des petits ruminants essential for interference and suppression of STAT-mediated interferon signaling. Virology.

[136] Chinnakannan S.K., Nanda S.K., Baron M.D. (2013). Morbillivirus v proteins exhibit multiple mechanisms to block type 1 and type 2 interferon signalling pathways. PLoS ONE.

[137] Abbas A.K., Lichtman A.H. (2003). Cellular and Molecular Immunology.

[138] Naik S., Renukaradhya G.J., Rajasekhar M., Shaila M.S. (1997). Immunogenic and protective properties of haemagglutinin protein (H) of rinderpest virus expressed by a recombinant baculovirus. Vaccine.

[139] Naik S., Shaila M. (1997). Characterization of Membrane-bound and Membrane Anchor-less Forms of Hemagglutinin Glycoprotein of Rinderpest Virus Expressed by Baculovirus Recombinants. Virus Genes.

[140] Sinnathamby G., Nayak R., Shaila M.S. (2001). Mapping of T-helper epitopes of Rinderpest virus hemagglutinin protein. Viral Immunol..

[141] Diallo A., Minet C., Le Goff C., Berhe G., Albina E., Libeau G., Barrett T. (2007). The threat of peste des petits ruminants: Progress in vaccine development for disease control. Vaccine.

[142] Rojas J.M., Rodriguez-Martin D., Avia M., Martin V., Sevilla N. (2018). Peste des Petits Ruminants Virus Fusion and Hemagglutinin Proteins Trigger Antibody-Dependent Cell-Mediated Cytotoxicity in Infected Cells. Front. Immunol..

[143] Mitra-Kaushik S., Nayak R., Shaila M.S. (2001). Identification of a cytotoxic T-cell epitope on the recombinant nucleocapsid proteins of Rinderpest and Peste des petits ruminants viruses presented as assembled nucleocapsids. Virology.

[144] Jones L., Giavedoni L., Saliki J.T., Brown C., Mebus C., Yilma T. (1993). Protection of goats against peste des petits ruminants with a vaccinia virus double recombinant expressing the F and H genes of rinderpest virus. Vaccine.

[145] Romero C.H., Barrett T., Chamberlain R.W., Kitching R.P., Fleming M., Black D.N. (1994). Recombinant Capripoxvirus Expressing the Hemagglutinin Protein Gene of Rinderpest Virus: Protection of Cattle against Rinderpest and Lumpy Skin Disease Viruses. Virology.

[146] Hu Q., Chen W., Huang K., Baron M.D., Bu Z. (2012). Rescue of recombinant peste des petits ruminants virus: Creation of a GFP-expressing virus and application in rapid virus neutralization test. Vet. Res..

[147] Muniraju M., Mahapatra M., Buczkowski H., Batten C., Banyard A.C., Parida S. (2015). Rescue of a vaccine strain of peste des petits ruminants virus: In vivo evaluation and comparison with standard vaccine. Vaccine.

[148] Niyokwishimira A., Dou Y., Qian B., Meera P., Zhang Z. (2018). Reverse Genetics for Peste des Petits Ruminants Virus: Current Status and Lessons to Learn from Other Non-segmented Negative-Sense RNA Viruses. Virol. Sin..

[149] Choi K.-S., Nah J.-J., Ko Y.-J., Kang S.-Y., Joo Y. (2003). Localization of antigenic sites at the amino-terminus of rinderpest virus N protein using deleted N mutants and monoclonal antibody. J. Vet. Sci..

[150] Renukaradhya G.J., Sinnathamby G., Seth S., Rajasekhar M., Shaila M.S. (2002). Mapping of B-cell epitopic sites and delineation of functional domains on the hemagglutinin-neuraminidase protein of peste des petits ruminants virus. Virus Res..

[151] Beckford A.P., Kaschula R.O., Stephen C. (1985). Factors associated with fatal cases of measles. A retrospective autopsy study. South Afr. Med. J. Suid. Afrik. Tydskr. Vir Geneeskd..

[152] Griffin D.E. (2010). Measles virus-induced suppression of immune responses. Immunol. Rev..

[153] Schneider-Schaulies S., Niewiesk S., Schneider-Schaulies J., ter Meulen V. (2001). Measles virus induced immunosuppression: Targets and effector mechanisms. Curr. Mol. Med..

[154] Schlender J., Schnorr J.J., Spielhoffer P., Cathomen T., Cattaneo R., Billeter M.A., ter Meulen V., Schneider-Schaulies S. (1996). Interaction of measles virus glycoproteins with the surface of uninfected peripheral blood lymphocytes induces immunosuppression in vitro. Proc. Natl. Acad. Sci. USA.

[155] Heaney J., Barrett T., Cosby S.L. (2002). Inhibition of in vitro leukocyte proliferation by morbilliviruses. J. Virol..

[156] Yoneda M., Miura R., Barrett T., Tsukiyama-Kohara K., Kai C. (2004). Rinderpest virus phosphoprotein gene is a major determinant of species-specific pathogenicity. J. Virol..

[157] Yoneda M., Bandyopadhyay S.K., Shiotani M., Fujita K., Nuntaprasert A., Miura R., Baron M.D., Barrett T., Kai C. (2002). Rinderpest virus H protein: Role in determining host range in rabbits. J. Gen. Virol..

[158] Yokota S., Saito H., Kubota T., Yokosawa N., Amano K., Fujii N. (2003). Measles virus suppresses interferon-alpha signaling pathway: Suppression of Jak1 phosphorylation and association of viral accessory proteins, C and V, with interferon-alpha receptor complex. Virology.

[159] Ravanel K., Castelle C., Defrance T., Wild T.F., Charron D., Lotteau V., Rabourdin-Combe C. (1997). Measles virus nucleocapsid protein binds to FcgammaRII and inhibits human B cell antibody production. J. Exp. Med..

[160] Avota E., Gassert E., Schneider-Schaulies S. (2010). Measles virus-induced immunosuppression: From effectors to mechanisms. Med. Microbiol. Immunol..

[161] Niewiesk S., Schneider-Schaulies J., Ohnimus H., Jassoy C., Schneider-Schaulies S., Diamond L., Logan J.S., ter Meulen V. (1997). CD46 expression does not overcome the intracellular block of measles virus replication in transgenic rats. J. Virol..

[162] Nizamani Z., Servan de Almeida R., Albina E., Parveen F., Libeau G. (2014). In vitro study of lymphotropic and immunomodulatory properties of the peste des petits ruminants virus (PPRV). J. Anim. Plant Sci..

[163] De Swart R.L., Ludlow M., de Witte L., Yanagi Y., van Amerongen G., McQuaid S., Yuksel S., Geijtenbeek T.B., Duprex W.P., Osterhaus A.D. (2007). Predominant infection of CD150+ lymphocytes and dendritic cells during measles virus infection of macaques. PLoS Pathog..

[164] Welstead G.G., Hsu E.C., Iorio C., Bolotin S., Richardson C.D. (2004). Mechanism of CD150 (SLAM) down regulation from the host cell surface by measles virus hemagglutinin protein. J. Virol..

[165] Erlenhoefer C., Wurzer W.J., Loffler S., Schneider-Schaulies S., ter Meulen V., Schneider-Schaulies J. (2001). CD150 (SLAM) is a receptor for measles virus but is not involved in viral contact-mediated proliferation inhibition. J. Virol..

[166] Romanets-Korbut O., Kovalevska L.M., Seya T., Sidorenko S.P., Horvat B. (2016). Measles virus hemagglutinin triggers intracellular signaling in CD150-expressing dendritic cells and inhibits immune response. Cell. Mol. Immunol..

[167] Sanz Bernardo B., Goodbourn S., Baron M.D. (2017). Control of the induction of type I interferon by Peste des petits ruminants virus. PLoS ONE.

[168] Hahm B., Cho J.H., Oldstone M.B. (2007). Measles virus-dendritic cell interaction via SLAM inhibits innate immunity: Selective signaling through TLR4 but not other TLRs mediates suppression of IL-12 synthesis. Virology.

[169] Caignard G., Bourai M., Jacob Y., Tangy F., Vidalain P.O. (2009). Inhibition of IFN-alpha/beta signaling by two discrete peptides within measles virus V protein that specifically bind STAT1 and STAT2. Virology.

[170] Boxer E.L., Nanda S.K., Baron M.D. (2009). The rinderpest virus non-structural C protein blocks the induction of type 1 interferon. Virology.

